# Can yesterday’s smoking research inform today’s shiftwork research? Epistemological consequences for exposures and doses due to circadian disruption at and off work

**DOI:** 10.1186/s12995-017-0175-4

**Published:** 2017-09-11

**Authors:** Thomas C. Erren, Philip Lewis

**Affiliations:** Institute and Policlinic for Occupational Medicine, Environmental Medicine and Prevention Research, University Hospital of Cologne, Cologne, Germany

**Keywords:** Smoking, Cancer, Shiftwork, Circadian disruption, Night work, Light, Chronobiology, Chronodisruption, Public health

## Abstract

In 1950, landmark epidemiology studies by Wynder & Graham and Doll & Hill contributed to identifying smoking as a potent carcinogen. In 2007, IARC classified shiftwork involving circadian disruption (CD) as probably carcinogenic; however, epidemiological evidence in regards to the carcinogenicity of shiftwork that involves nightwork is conflicting.

We hypothesize that shiftwork research is lacking chronobiological and methodological rigor and that lessons can be learned from comparison with smoking research. Herein, we provide a factual view at, and a fictional case study of, 1940s smoking research which serves as an analogy for current shiftwork research dilemmas. This analogy takes the form of limiting counting cigarettes to a particular time window (i.e. at work) rather than assessing exposures to, and doses of, accumulated smoking over 24 h, highlighting the importance of exposure and dose. Simply put, smoking insights could have been delayed or even disallowed.

In conclusion, CD may be similar to smoking insofar as for quantitative measures of cumulative doses, exposures both at and off work may have to be considered. Future work must explore whether such similarity factually exists and whether CD is a cancer hazard in IARC terms.

*“Epidemiology is certainly a poor tool for learning about the mechanism by which a disease is produced, but it has the tremendous advantage that it focuses on the diseases and the deaths that actually occur, and experience has shown that it continues to be second to none as a means of discovering links in the chain of causation that are capable of being broken.”* -Sir Richard Doll [1]

## Introduction

In 2007, the International Agency for Research on Cancer (IARC) classified shiftwork involving circadian disruption (CD) as probably carcinogenic [2, 3]; however, epidemiological evidence in regards to the carcinogenicity of shiftwork that involves nightwork is conflicting [4–15]. We hypothesize that shiftwork research is lacking chronobiological and methodological rigor and that lessons can be learned from comparison with smoking research. Herein, we provide a factual view at, and a fictional case study of, 1940s smoking research which serves as an analogy for current shiftwork research dilemmas regarding CD exposure and dose.

## A factual view at smoking research

In the early twentieth century, lung cancer was a rare disease but suspicion of increasing incidence was taking hold. In answering whether the lung cancer increase was real [16, 17] rather than an artefact [18, 19], two questions were grappled with [1]: Could lung cancer have gone unnoticed in people who had died at significantly younger ages before the 1900s’ improved hygiene and medicine allowed an increased life expectancy? Could the increased number of lung clinics in response to rising cancer numbers or improved diagnostic tools such as chest x-rays have fueled better detection rather than mirror more frequently occurring disease? Once detection bias was ruled out, key hypotheses to explain the observations included air pollution and smoking [20]. That the lung cancer increase affected men rather than women made the air pollution hypothesis less persuasive. Conversely, that smoking was a much more prevalent vice in men than in women meant smoking research became urgent. In 1950, two studies from the UK [21] and the US [22] provided strong evidence that smoking was the long-sought lung cancer culprit. However, smoking remained a controversial topic in the second half of the twentieth century with eminent statisticians such as Fisher and Berkson opposing the idea that cigarette smoking causes cancer [1]. Continuous academic controversies served to advance study methodology including how to pass from statistical associations to verdicts of causation. As a key achievement, smoking research provided both the incentive and the topic to develop what is known as Hill’s viewpoints which are used when weighing observed evidence for or against causality between exposure, dose, and disease [23].

## A fictional case study of smoking research

Reflecting on the remarkable research of Doll and Hill [21], his own contribution [22], and contributions of others in that period [24], Wynder referred to “dose response” and “sound biological reasons” as aspects that facilitated epidemiological breakthroughs in the late 1940’s:

*“As it was, a patient’s history of cigarette smoking was quite easy to obtain. There was a group that never smoked, and there were sound biologic reasons to assume a causative relation. The relative risks were so large that, in fact, our paper published in 1950* [22] *included no statistical testing.”* [24]

Let us imagine an alternative course of events, viz. what might have happened if the “sound biological reasons” had been misinterpreted and smoking habits erroneously classified. For instance, what if smoking at work – possibly due to interactions with one or more workplace factors such as arsenic [25], asbestos [26], or ionizing radiation [27] – had been mistaken for the exclusive cancer culprit which needed testing? In other words, what if epidemiologists had hypothesized erroneously that only smoking at work was a “component cause” [28] of cancer rather than all smoking combined? Table 1 exemplifies fictional misclassifications (alongside the factual classification [22]) if smoking had been assessed at work alone in contrast to over the entire day. Clearly, ignoring cigarettes smoked off work could have completely masked, or significantly attenuated, risk estimates attributed to smoking as a synergistic cancer factor with asbestos (for example) at work [26]. Equally clearly, it can be predicted that smoking insights could have been delayed or disallowed had research failed to assess exposures to, and arrive at doses of, cigarette smoking at and off work cumulatively.

**Table 1:** Factual classification of smoking habits in Wynder and Graham, 1950, [22] and fictional misclassification

Factual Wynder & Graham 1950			Fictional smoking distribution		
Individual	Cigarettes^a^	Classification	Cigarettes^a^		Mis-classification
	Cumulative		At work	Off work	
A	<1	Nonsmoker	–	<1	–
B	1–9	Light smoker	0	1–9	Nonsmoker
C	10–15	Moderately heavy smoker	5	10	Light smoker
D	16–20	Heavy smoker	0	16–20	Nonsmoker
E	21–34	Excessive smoker	5	16–29	Light smoker
F	≥35	Chain smoker	10	≥25	Moderately heavy smoker

Note that the fictional smoking distribution does not represent any descriptive statistics regarding smoking at or off work in 1950

^a^per day for more than 20 years

This fictional example illustrates how erroneous classifications of smoking could have been misleading if focused on work *alone*. Epistemologically, it highlights the necessity to appropriately assess exposure and dose. Thankfully, in the 1940s, researchers obtained relevant exposure and dose gradients by simply asking study individuals how many cigarettes they smoked and for how long. Indeed, such information derived by the number of cigarettes smoked per day and the number of years for which individuals had smoked (“pack-years”) was both necessary and sufficient to unmask smoking as the cancer cause.

## When yesterday’s smoking research meets today’s shiftwork research

With the appreciation of dire implications of our fictional smoking research scenario from the last century, let us look at the current state of research into shiftwork and disease, including cancer. In 2007, after diligent review of the published evidence, 22 IARC experts concluded that “shift-work that involves circadian disruption is probably carcinogenic to humans”. The working group based their Group 2A classification on “sufficient evidence in experimental animals for the carcinogenicity of light during the daily dark period (biological night)” and on “limited evidence in humans for the carcinogenicity of shiftwork that involves nightwork” [2, 3].

Given that smoking research is compatible with the following postulates

- suspected culprits must be assessed in full i.e. the culprit should be captured whenever individuals are exposed
- wherever possible, we must arrive at doses and should not confine our studies to exposure alone [29, 30]

shiftwork research must answer the following questions:

- When do individuals experience the probable cancer culprit (exposure)?
- How much are individuals exposed to the probable cancer culprit (dose)?

From the IARC conclusion it is clear that shiftwork *that involves CD* is the probable carcinogenic culprit. However, one possible way to misinterpret the IARC conclusion would be to attribute probable carcinogenicity to CD resultant from light, or other circadian challenges, during the daily dark period specifically rather than resultant from such exposures during an individual’s biological night (BN). Chronobiologically, it is not work at night but work and other activities at the BN which epidemiologists need to target when examining probable links between resulting CD and cancer. A second possible misconception would be to focus research on, and to limit it to, CD exposure rather than CD dose.

Similar to the 1940s–smoking research, proper interpretation of exposure and dose is essential to answer the key question: How can much-needed epidemiological research explore whether shiftwork involving CD is factually carcinogenic to humans or not? To establish accurate exposure, we must first determine “what” and “when” individuals’ BNs and biological days (BDs) are. At the core of IARC’s 2007 classification lies disrupted chronobiology. Chronobiology is genetically (co-)determined [31, 32] and can be delineated into BDs (periods when one is primed for activity) and BNs (periods when the propensity is to sleep). On chronobiological grounds, CD can be expected if work or activities (and associated light exposures) are carried out (or experienced) when the body is prepared for, and anticipates, sleep. And therein we also have a more appropriate assessment of exposure, viz. activities both at and off work during the BN (excluding sleep) rather than at work *alone*. “Sound biological reasons” [24] suggest that such CD, disrupting the circadian organization of physiology, endocrinology, metabolism, and behavior, may lead to cancer and possibly other diseases [33]. With this background, it seems imperative to consider the following aspects when designing epidemiological studies to assess CD and shiftwork:

- Chronobiological propensities for wake and sleep vary between individuals. While we lack precise percentages across populations, humans can be grouped into early [“lark”], intermediate, and late [“owl”] chronotypes. Chronotype-specific activity periods may extend into the daily dark period and chronotype-specific sleep periods may extend into the daily light period. Therefore, simply assessing work per se during the dark period is not only insufficient, it is potentially erroneous.
- To assess an individual’s total BN-associated CD, in addition to work during BNs activities off-work during BNs must be operationalized.

In contrast to what is required to assess CD, all 39 epidemiological studies into cancer risks after IARC 2007 compared risks between shiftworkers and non-shiftworkers or between night and day workers without specifically targeting CD (Table 2). While 9 studies [11, 34–41] collected information regarding chronotype or chronobiological propensity, none of these took note of the BN as the vulnerable time window in their analyses. Furthermore, none of the post-IARC 2007 studies took BN-activities off work into account. Disconcertingly, while these data may be employed for “traditional” shiftwork research, these data cannot be used for interpretable explorations of hypotheses regarding the carcinogenicity of shiftwork that causes, or is associated with, CD. This confinement of research into the effects of CD may unfortunately be analogous with the fictional case study of smoking research presented above. In other words, confining shiftwork epidemiology to the civil night [42] and assuming that CD does not occur in other time windows may be similarly deceiving as confining studies to “smoking at work” and ignoring effects due to “smoking off work”. Taken together, none of the 39 epidemiological studies after IARC assessed cumulative CD doses due to activities both at and off work.

**Table 2:** Pre- and Post-IARC 2007 studies of shift work and cancer: Targeted assessment of chronotype, internal time, or circadian disruption

Publication	Targeted assessment				
First author, year	Cancer Endpoint	Chronotype/Chronobiological propensity	Biological day or Biological night	Circadian disruption	
				At work	Off work
Pre-IARC					
Tynes, 1996 [56]	Breast	–	–	–	–
Davis, 2001 [57]	Breast	–	–	–	–
Hansen, 2001 [58]	Breast	–	–	–	–
Schernhammer, 2001 [59]	Breast	–	–	–	–
Schernhammer, 2003 [60]	Colorectum	–	–	–	–
Lie, 2006 [61]	Breast	–	–	–	–
O’Leary, 2006 [62]	Breast	–	–	–	–
Schernhammer, 2006 [63]	Breast	–	–	–	–
Kubo, 2006 [64]	Prostate	–	–	–	–
Schwartzbaum, 2007 [65]	Cancer	–	–	–	–
Viswanathan, 2007 [66]	Endometrium	–	–	–	–
Conlon, 2007 [67]	Prostate	–	–	–	–
Post-IARC					
Lahti, 2008 [68]	Non-Hodgkin	–	–	–	–
Pukkala, 2009 [69]	Cancer	–	–	–	–
Pesch, 2010 [70]	Breast	–	–	–	–
Pronk, 2010 [71]	Breast	–	–	–	–
Kubo, 2011 [72]	Prostate	–	–	–	–
Schernhammer, 2011 [73]	Skin	–	–	–	–
Lie, 2011 [74]	Breast	–	–	–	–
Hansen, 2012a [34]	Breast	Yes	–	–	–
Hansen, 2012b [75]	Breast	–	–	–	–
Parent, 2012 [76]	Cancer	–	–	–	–
Knutsson, 2013 [77]	Breast	–	–	–	–
Menegaux, 2013 [78]	Breast	–	–	–	–
Rabstein, 2013 [79]	Breast	–	–	–	–
Bhatti, 2013 [35]	Ovary	Yes	–	–	–
Lin, 2013 [80]	Pancreas	–	–	–	–
Grundy, 2013 [81]	Breast	–	–	–	–
Fritschi, 2013 [36]	Breast	Yes	–	–	–
Schernhammer, 2013 [82]	Lung	–	–	–	–
Grundy, 2013 [83]	Breast	–	–	–	–
Gapstur, 2014 [84]	Prostate	–	–	–	–
Carter, 2014 [85]	Ovary	–	–	–	–
Koppes, 2014 [86]	Breast	–	–	–	–
Truong, 2014 [87]	Breast	–	–	–	–
Yong, 2014 [88]	Cancer	–	–	–	–
Rabstein, 2014 [89]	Breast	–	–	–	–
Kwon, 2015 [90]	Lung	–	–	–	–
Li, 2015 [91]	Breast	–	–	–	–
Akerstedt, 2015 [92]	Breast	–	–	–	–
Hammer, 2015 [93]	Prostate	–	–	–	–
Papantoniou, 2015 [37]	Prostate	Yes	–	–	–
Lin, 2015 [94]	Biliary tract	–	–	–	–
Cordina-Duve., 2016 [95]	Breast	–	–	–	–
Gyarmati, 2016 [38]	Stomach	Yes	–	–	–
Papantoniou, 2016 [39]	Breast	Yes	–	–	–
Travis, 2016 [11]	Breast	Yes	–	–	–
Costas, 2016	CLL	–	–	–	–
Dickerman, 2016 [40]	Prostate	Yes	–	–	–
Heckman, 2017 [41]	Skin	Yes	–	–	–
Papantoniou, 2017 [96]	Colorectum	–	–	–	–

## Resolving issues of exposure and dose

Generally speaking, answers to both (a) and (b) above can be provided by basic chronobiology and/or the CD-related concept of chronodisruption [43], operationalized as the split physiological nexus of internal and external times [44], which can allow epidemiological studies of shiftwork involving CD and cancer. Such split or disrupted nexus may be indicated or caused by activities during individuals’ BNs and/or sleep during their BDs.

More specifically, to answer (a) researchers may assess whether individuals are exposed to CD by comparing activities at and off work during the individuals’ BNs and/or sleep during their BDs. Answers to (b) can be provided by summing up over years or decades, how much working times overlap with an individual’s BN or how much sleep times overlap with their BDs (Table 3). The resulting CD_hours_ may – at least in theory – yield significant doses of CD in certain chronotypes who were never engaged in so-called nightshifts. This has been shown through simple summations [45].

**Table 3:** Factual smoking assessment and proposed assessment of circadian disruption as activities during BNs or sleep during BDs

	Targeted assessment				
	Smoking		Circadian disruption		
	Cigarettes/24 h		Activities/BN		Sleep/BD
Exposure	At work	Off work	At work	Off work	na
	yes	yes	yes	yes	na
Dose	# of cigarettes	# of cigarettes	# of activity hours/BN		# of sleep hours/BD
	Cumulative Cigarettes		Cumulative CD_BN_hours		Cumulative CD_BD_hours

In practice, answering (a) and (b) will be much more demanding than asking study participants for their history of cigarette smoking. Assessing how much individuals smoked during their lifetime as a basis for exposures and doses was straightforward, viz. cigarettes smoked at any time needed simple counting. Quite differently, assessing exposure to, and doses of, CD is complex as individuals’ activities both at and off work, or sleep, need to be compared in regards to their overlap with individuals’ BNs or BDs. As a prerequisite for (a) and (b), we need information for internal or biological time. This may be approximated by questionnaires such as the morningness-eveningness questionnaire (MEQ) [46], the MunichChronoType Questionnaire (MCTQ) [47], or the perfect day approach [48]. Establishing when individuals worked, what activities they engaged in when off work, and to what extent these times overlapped with their BN may pose significant challenges. It may thus be easier to assess the counterpart of activities at and off work during the BN, viz. how much do the time windows of sleep overlap with individual BDs. To this end, the proposed sleep-years index [49, 50] could be extended to a sleep-time window assessment which asks in what time windows (i.e. when and how long) study participants regularly slept/sleep (retrospective/prospective studies). Similar to the pack-years concept, information on average hours of sleep in relevant time windows could be collected. In retrospective studies, decisive and memorable events in life such as graduation, marriage, pregnancy, caring for children, employment changes, personal losses, grief, illness, stress or anxiety, and so on may help to recall and mark points and periods in life that may be associated with changes of both the duration [51] and timing of sleep over decades.

Information provided through such sleep-time window assessment could be used in two ways: First, the accumulated hours of sleep during the BD over many years could approximate what has been called “accumulated sleep disruption” (ASD) [52] which could be utilized as a proxy for BD-associated total CD, i.e. having to sleep during the individual BD due to activities at and off work; Second, activity times could be approximated from the factually reported sleep-times to yield CD_BN_, albeit indirectly without asking for activity times at and off work. Likewise, sleep-time windows may be approximated from factually reported activity times. Ultimately, if direct questionnaire information were available on both the activities at and off work, on the one hand, and on the sleep-time windows, on the other, risk estimates associated with directly and indirectly computed CD_BN_ or CD_BD_ could be compared.

Epistemologically, although studies investigating adverse health effects, including cancer, of total or cumulative CD will be challenging in practice, they may be without alternatives. This corresponds with the viewpoints offered by Hill in 1965 – “from all of which we should study association before we cry causation”. When addressing “viewpoint (5) = biological gradient”, the protagonist of smoking and causality research captured what may be at stake with regard to CD:

*“Often the difficulty is to secure some satisfactory quantitative measure of the [cause-in-question] which will permit us to explore …. dose-response. But we should invariably seek it.”* [23]

## Perspectives

Clearly, we challenge the expectation that CD caused by work is the exclusive source of a total dose of CD. Epistemologically, CD off work – similar to smoking off work – could be part of the cumulative or total CD dose. For CD at and off work to be summed up, a chronobiological prerequisite will be to consider individuals’ biological time, a difficult venture on its own. Equally clearly, we must avoid the erroneous notion that shiftwork can be confused with “the new smoking”. Regarding quantitative dose measures, there may be a similarity between CD and smoking insofar as CD – like smoking – at and off work may be pathophysiologically relevant. Only future work may demonstrate whether such similarity regarding cumulative doses exists and whether total CD is a hazard at and off work in IARC terms. At this stage, answers to what extent CD at and off work may be associated with disease, including cancer, are completely speculative and must be avoided.

Similar to our lack of knowledge regarding possibly synergistic actions of CD and workplace factors, we do not know how CD caused by activities at and off work during the BN can be compared. In addition to being experienced at a biologically unfavorable time, different stimuli may elicit different intensities of CD. For example: alcohol, coffee, food, dancing, manual labor, or sitting at a desk may differentially intensify or lessen CD dose. Light exposures, with their complex role on sleep and CD being increasingly considered [53], during work and activities off work are expected to play a key role.

How we suggest quantifying CD associated with activities at and off work during the BN may require some weighting. Indeed, neither we nor “traditional” shiftwork epidemiology know how consecutive shifts or activities during the BN affect CD and how adaptation to chronodisruption may require modified assessments of CD rather than simply adding up CD_BN_hours [45]. Moreover, whether CD_BN_ and CD_BD_ generated by, or associated with, activities during the BNs or sleep during the BDs can be comparable in regards to possible effects leading to disease, including cancer, is open. Nonetheless, to begin to understand causal networks that involve CD, we suggest to compare chronodisruption caused/indicated by activities – at and off work – during individuals’ biological nights with chronodisruption caused/indicated by (the more amenable information of) sleep during individuals’ biological days.

Taken together, while research in the workplace may have first pointed to adverse health effects of work during the BN, CD could be a relevant consequence of behavior both at and off nominal work. In this vein, shiftworkers may be viewed as sentinels or indicators of a causal phenomenon, viz. CD, which can affect humans in different susceptible time windows over 24 h and may contribute to a so-called background incidence of disease, including cancer, in the general population. If we continue with “traditional” shiftwork research i.e. if we confine our search for a probable carcinogenic culprit to nominal night-shifts or nominal shiftwork, we may miss both the existence and the magnitude of effects associated with CD. Critically, as long as the relevance of the biological time concept is not falsified, ignoring both variable biological nights in individuals and variable sources of CD at and off work may explain why “traditional” shiftwork research fails to detect risks which numerous people expect from activities and behavior at chronobiologically unusual times [4].

## Conclusions

Epistemologically, current shiftwork epidemiology lacks chronobiological and methodological rigor because CD has been improperly and inadequately assessed. The consequences of this may be significant as per the fictional case study of smoking epidemiological research presented above and that data collected so far disallowing to explore hypotheses regarding the carcinogenicity of “shiftwork that involves CD” [2, 3].

Researchers may argue that proposing to assess CD caused by activities at [54] and off work during individuals’ biological night and/or sleep during the biological day to explore adverse health effects of disturbed chronobiology is easy to demand but hard to do. They are right [55]. However, we are faced with the fact that IARC experts identified shiftwork involving CD as “probably” carcinogenic to humans. Looking at the magnitude of exposed individuals and the impact of suspected endpoints such as cancer of the breast and prostate, it is an ethical must to solve the CD riddle at the workplace – and beyond.

In conclusion, while the analogy with “fictional” consequences of smoking research may appear extreme, the biological and methodological rigor of smoking research should teach us lessons; namely, to comprehensively identify CD exposures, to strive to estimate CD doses, and to be prepared that reliable tools to assess the latter may be(come) a *conditio* sine qua non to elucidate causal links of CD with cancer and a host of other diseases [33].

## Abbreviations

ASD: Accumulated sleep disruption
BD: Biological day
BN: Biological night
CD: Circadian disruption
IARC: International Agency for Research on Cancer
MCTQ: Munich Chronotype Questionnaire
MEQ: Morningness-eveningness questionnaire
